# Being on Your Own or Feeling Lonely? Loneliness and Other Social Variables in Youths with Autism Spectrum Disorders

**DOI:** 10.1007/s10578-016-0707-7

**Published:** 2017-01-09

**Authors:** Anne Deckers, Peter Muris, Jeffrey Roelofs

**Affiliations:** Virenze RIAGG Maastricht and Department of Clinical Psychological Science, Maastricht University, Post box 616, 6200 Maastricht, MD The Netherlands

**Keywords:** Loneliness, Autism spectrum disorders, Children vs. adolescents, Desire for social interaction

## Abstract

A cross-sectional study was conducted to examine loneliness and its correlates in children (7 to 11 years) and adolescents (12 to 18 years) with autism spectrum disorders (ASD, *n* = 73) and control groups of clinically referred (ADHD, *n* = 76) and non-clinical (*n* = 106) youths. Youths completed questionnaires on loneliness and desire for social interaction, while parents and teachers filled out scales on other aspects of children’s social functioning. Results indicated that only at an adolescent age, the ASD group reported higher levels of loneliness than the control groups. Further, the ASD group generally expressed relatively low levels of desire for social interaction, although these youths displayed a similar increase in the wish to belong during adolescence as participants in the control groups. Finally, the ASD group exhibited lower levels of social competence and social skills and higher levels of social problems and social anxiety than the control groups, and in all groups these social variables correlated in a theoretically meaningful with loneliness.

## Introduction

The need to belong is an essential characteristic of human beings and a strong motive guiding our cognitions, emotions, and behavior [1]. Although from an evolutionary perspective the importance of belonging nowadays might be less crucial for survival and reproduction as compared to our ancestors, it is still thought to be a critical factor determining people’s emotional well-being [2]. When the need to belong is not satisfactorily met, feelings of loneliness may arise [3]. Loneliness is a subjective and negative experience [4] that derives from a discrepancy between the interpersonal relationships people desire and the quantity and quality of these relationships that are actually perceived [5].

Loneliness involves intra-individual characteristics like self-esteem and shyness as well as inter-individual experiences referring to positive and negative peer interactions varying from social acceptance and friendship to bullying and victimization [6]. Loneliness appears to be the result of a complex interplay between a person’s desires, social abilities, perceptions, and interpretations, and social interactions and thus reciprocal processes with others. While it is perfectly normal to feel lonely every now and then, it is also clear that persistent and increased feelings of loneliness have to be considered as clinically relevant. This is supported by research findings demonstrating that loneliness is associated with mental health problems, like anxiety disorders and depression, and even physical complaints and diseases [3].

Loneliness is a prevalent phenomenon during adolescence and can be considered a common experience in this developmental phase [3]. Adolescents might be more susceptible to feelings of loneliness for several reasons. During adolescence youths tend to shift from family to peers as their most important companions. While they become more individuated and autonomous towards their parents and involvement in the peer group becomes more important, they also have to handle their pubertal maturation and their identity formation process [7]. Social interactions become more complicated, and the awareness of social competence of the self and others increases. During this transition, it might be quite difficult to fulfil their expectations about social relationships and to satisfy their need to belong resulting in loneliness at some point [3]. For youngsters with autism spectrum disorders (ASD) it might be particularly difficult to keep up with their peers and to be involved with and accepted by them.

Deficits in communication and problems with social interaction are core characteristics of children and adolescents with ASD [8, 9]. For example, youth with ASD have less social skills [10], are more often victim of bullying [11], experience poorer friendship quality [12], spend less time interacting with others [13], and have a less central position in social networks [14, 15]. Given these social difficulties, one might expect that children and adolescents with ASD are at increased risk for experiencing feelings of loneliness. Several studies have shown that children and adolescents with ASD indeed report higher levels of loneliness relative to their non-clinical peers [14, 16–20]. However, Chamberlain and colleagues [21] did not find elevated levels of loneliness for younger children with ASD as compared to typically developing control children. It has been suggested that the experience of loneliness in youth with ASD might be age-dependent and becomes more pronounced during adolescence [19]. In fact, previous studies that documented elevated levels of loneliness in relation to ASD mainly relied on samples that predominantly included adolescents [14, 18–20]. Besides that, the sample sizes of these studies were often relatively small, and so in studies that did include younger children it was not possible to explore the experience of loneliness in youths with ASD of various ages [16, 17].

Meanwhile, some scholars have suggested that youths with ASD have less desire for social interaction [22] or a stronger wish to be on their own [23], and as such children and adolescents with ASD might be less prone to develop feelings of loneliness. However, it should be noted that a stronger desire for aloneness and loneliness are not mutually exclusive [3, 24]. In addition, previous research assessing the desire for social interaction in 7- to 12-year-old children with ASD demonstrated that these youngsters did show a desire for social interaction on an implicit level (as measured by means of an experimental approach-avoidance task), while they expressed less desire for social interaction on an explicit level (as assessed with a questionnaire) as compared to typically developing children [25]. It might be that social deficits hinder youths with ASD to openly express their desire for social interaction. That is, it is possible that they do experience a desire for social interaction or a need to belong, but lack the skills and/or need specific circumstances in order to be able to translate this wish into action.

It is unclear whether increased levels of loneliness are specific for ASD or are more generally related to developmental disorders or psychopathology in youths [19]. As mentioned before, loneliness is related to different types of psychopathology like depression and anxiety, but the direction of causality is still ambiguous and might also be bi-directional [3, 26]. However, for other types of psychopathology loneliness is more likely to be a consequence rather than cause of the symptomatology. For example, it could be possible that youths with Attention Deficit Hyperactivity Disorder (ADHD) experience higher levels of loneliness, but loneliness alone is unlikely to cause ADHD. This makes youth with ADHD an interesting clinical comparison group, although it should be admitted that the studies conducted so far have yielded quite mixed results. Heiman [27] documented increased levels of parent- and teacher-rated loneliness, but not on self-reported loneliness when comparing an ADHD group with a non-clinical control group. However, in a more recent study, Houghton and colleagues [28] found comparable levels of loneliness in children and adolescents with ADHD and their typically developing counterparts.

In the present study we took a closer look at the phenomenon of loneliness in youths with ASD, adopting a multi-informant approach using assessments of children themselves, parents, and teachers. Three groups were included in this investigation: an ASD group, a clinical control group of youths with ADHD, and a typically developing control group. Each group consisted of children (i.e., age 7–11 years) as well as adolescents (i.e., age 12–18 years). It was hypothesized that (1) the ASD group would show increased levels of loneliness as compared to the typically developing and the clinical control group, and (2) adolescents would display higher levels of loneliness than children. In addition to loneliness, we also measured youths’ desire for social interaction, social skills, social problems, social competence, and social anxiety, which enabled us to explore their relation with loneliness. For these other social variables, we also made group comparisons. Here we expected to find (3) lower levels of desire for social interaction, lower levels of social skills and social competence, and higher levels of social problems and social anxiety in the ASD group as compared to both control groups. Our predictions regarding differences between the clinical and non-clinical control groups were less clear, but on the basis of what has been found in the literature it might be expected that children and adolescents with ADHD would show lower levels of social skills and social competence, and higher levels of social problems than their typically developing counterparts [29–31]. Finally, the relations between loneliness and the other social variables were explored. Here it was hypothesized that (4) loneliness would correlate positively with desire for social interaction, social problems, and social anxiety, and negatively with social skills and social competence.

## Method

### Design and Procedure

A cross-sectional research design was applied using multiple informants. Children (7–11 years) and adolescents (12–18 years) with ASD were compared to clinical and non-clinical control groups of children and adolescents. The participants in the typical control group were recruited from regular primary and secondary schools in the Netherlands. The participants in the ASD group and the clinical control group were recruited at the community mental health center (Virenze-RIAGG Maastricht, The Netherlands). For the ASD group, the main inclusion criterion was a formal diagnosis of Autistic Disorder, Asperger’s Disorder, or Pervasive Developmental Disorder-Not Otherwise Specified (PDD-NOS) in terms of the *Diagnostic and Statistical Manual of Mental Disorders, fourth edition, text revision* (DSM-IV-TR) [8]. For the clinical control group, a formal diagnosis of Attention-Deficit/Hyperactivity Disorder was required, whereas suspicion of autism spectrum problems was an exclusion criterion. For both the ASD and clinical control group the presence of comorbid (psychiatric) diagnoses was allowed. The clinical diagnosis was based on an extensive diagnostic procedure according to the longitudinal-expert-all data (LEAD) principle [32]. That is, the diagnoses were made by a multidisciplinary team including licensed psychiatrists and psychologists, using information from multiple sources (i.e., interviews with the child, parents and teacher, psychiatric examination, psychological assessment, and clinical observations) during a longitudinal diagnostic and/or treatment process. Children with severe cognitive (i.e., estimated IQ <70) or language impairments were not included in the study. Parents and teachers of the children and adolescents in all three groups were also invited to participate. The study was approved by the Ethical Committee of Psychology at Maastricht University and participation only occurred after written informed consent was given. Parents gave informed consent for their own participation in the research and for the participation of their child. All children were informed about the research and children aged 12 years and older also had to sign an informed consent form. Teachers were only invited to participate after parents had given written permission for an assessment via this informant. If this was the case, teachers received information about the study and eventually gave consent for their contribution.

### Participants

The total sample consisted of 255 children and adolescents: 73 in the ASD group (47 children and 26 adolescents), 76 in the clinical control (ADHD) group (36 children and 40 adolescents), and 106 in the non-clinical control group (54 children and 52 adolescents). Complete assessments of the child via all three informants were conducted in 66% of the cases. In other cases, only parent- and self-report (24%), teacher- and self-report (4%), or self-report (3%) measurements were carried out. Table 1 shows descriptive characteristics for each of the three groups. In accordance with epidemiological studies, skewed gender differences were found in both clinical groups [33, 34]. Thus, in the ASD and the clinical control groups, there were clearly more boys than girls, whereas in the non-clinical group both genders were more equally present [χ^2^(2, 255) = 14.42, p = .001]. The mean age of the total sample was 11.6 years (*SD* 2.55) and age did not differ among the three groups [*F*(2,255) = 1.00, *p* = .37]. The sample consisted predominantly of Caucasian participants. In the ASD group, about 73% of the participants attended regular education, whereas 27% were in special schools. In the clinical control group, 89% of the children and adolescents followed regular education and 11% were in special education.

**Table 1:** Descriptive characteristics (gender and age distributions) of the total sample and each of the three groups

Descriptive characteristics	Total sample (*N* = 255)	ASD group (*n* = 73)	Clinical control (*n* = 76)	Non-clinical control (*n* = 106)
Male/female (numbers)				
Total	178/77	62/11	54/22	62/44
Child	104/33	42/5	26/10	36/18
Adolescent	74/44	20/6	28/12	26/26
Age (*M, SD*)				
Total	11.55 (2.53)	11.22 (2.42)	11.79 (2.48)	11.61 (2.63)
Child	9.56 (1.17)	9.81 (1.06)	9.61 (1.13)	9.31 (1.26)
Adolescent	13.86 (1.50)	13.77 (2.07)	13.75 (1.55)	14.00 (1.10)

In the child ASD group, 63.8% was diagnosed with PDD-NOS, 25.5% with Asperger’s Disorder, and 8.5% with Autistic Disorder. In the adolescent ASD group, the distribution was comparable: 69.2% was diagnosed with PDD-NOS, 19.2% with Asperger’s Disorder, and 11.5% with Autistic Disorder. The majority of the child clinical control group was diagnosed with ADHD of the combined subtype (81%), while the other children had ADHD of the inattentive subtype (19%). In the adolescent clinical control group, 75% was diagnosed with ADHD of the combined subtype, 22.5% with the inattentive subtype, and 2.5% with the hyperactive-impulsive subtype. In both clinical groups, comorbidity rates were equally high [χ^2^(1, 149) <1] (see Table 2). In the ASD group, 55% of the youths were diagnosed with at least one comorbid diagnosis, while 23% even met the criteria for two or more comorbid conditions. In the clinical control group, these figures were respectively 62% and 33%.

**Table 2:** Overview of the frequencies (percentages) of comorbid diagnoses in the two clinical groups

Comorbid clinical diagnosis	ASD group (*n* = 73)	Clinical control (*n* = 76)
No comorbid diagnosis	33 (45.2)	29 (38.2)
Adjustment disorders	1 (1.4)	4 (5.3)
ADHD	17 (23.3)	*
Anxiety disorders	9 (12.3)	5 (6.6)
Disruptive behavior disorders	0 (0.0)	6 (7.9)
Eating disorders	2 (2.7)	0 (0.0)
Identity/personality problems	0 (0.0)	3 (3.9)
Learning disorders	4 (5.5)	18 (23.7)
Mood disorders	1 (1.4)	4 (5.3)
Relational problems	23 (31.5)	34 (44.7)
Somatoform disorders	0 (0.0)	2 (2.6)
Other disorder or diagnosis deferred	2 (2.7)	9 (11.8)

*All youths in the clinical control group had a diagnosis of ADHD

### Questionnaires

The *Louvain scale of Loneliness and Aloneness in Children and Adolescents* (LACA; [35]) consists of four subscales: Loneliness in the relationships with parents, loneliness in the relationships with peers, aversion to aloneness, and affinity for aloneness. In the present study, only the loneliness in the relationships with peers subscale was used and regarded as the primary measure. This subscale contains 12 items (e.g., “I think I have fewer friends than others”) for which participants indicate how often the item applies to them using a Likert scale ranging from never (1) to often (4). A total score can be calculated ranging between 12 and 48, with higher scores indicating higher levels of loneliness in the relationships with peers. The internal consistency of the LACA has been shown to be high and its validity is satisfactory in large samples of elementary-school children and adolescents [36, 37]. The Cronbach’s alpha of the LACA subscale that was employed in the current study was also good (α = 0.88).

The desire for social contact with others was assessed by means of the *Wish for Social Interaction Scale* (WSIS; [25]). The WSIS contains 64 items referring to possibly conducting future social activities with unfamiliar individuals. Participants are asked whether they want to engage in 8 different social activities with 8 unknown persons by asking questions such as “Would you like to have a little chat with this person?” or “Would you like to play with this person?” Each item is accompanied by a side-view picture of the face of an unknown person displayed on a computer screen. Each question has to be answered on a yes/no format, and thus a total score can be calculated by summing the number of positive responses (range 0–64). The child and adolescent participants in the present study received similar questions. The pictures of the (male and female) adult faces were the same for both groups, but the pictures of the peers were adjusted for each age group and thus different. The Cronbach’s alpha of the WSIS was found to be excellent in the current study (α = 0.93) which is comparable to what was found in our previous study [25]. Further, pilot data collected in our own lab show that youths’ WSIS scores are substantially correlated (*r* = .56) with the Desire for Future Interaction Scale (DFI; [38]), which provides support for the validity of the scale.

The level of social skills was assessed using the *Social Skills Observation* (SSO; [39]), which was completed by both parents and teachers. The SSO asks parents and teachers to evaluate the behavior of the children and adolescents in various types of social situations during the past week: greeting, conversation, and play. Respondents have to indicate whether or not the child or adolescent engaged in these types of social interactions with other persons. In case that this is indeed the case, additional questions are asked pertaining to the specific social skills displayed by the child/adolescent (e.g., “Did he/she make eye contact?”; 36 items). Subsequently, 7 items about more general social skills are asked (e.g., “Did he/she remain at an appropriate distance from the other person?”). The SSO was slightly adapted for the use in the adolescent participants. That is, the items referring to “play” were altered in “joint activity”. A total score reflecting the level of social skills can be calculated by summing the “yes” responses. In the current study, the internal consistency of both the parent (α = 0.93) and teacher (α = 0.92) version of the SSO was excellent. The correlation between the parent and teacher version of the SSO was moderate (*r* = .26), but nevertheless supports the validity of the scale.

The *Achenbach System of Empirically Based Assessment* [40] was used to assess the level of social problems and social competence. The Social Problems and Social Competence scales of the Child Behavior Checklist (CBCL) were filled out by the parents, while teachers completed the Social Problems subscale of the Teacher Report Form (TRF). The Social Problems subscale of both the CBCL and the TRF consists of 11 items which have to be rated on a 3-point scale (0 = not true, 1 = somewhat or sometimes true, and 2 = very true or often true). One of the items pertains to “Complains of loneliness”, and in order to prevent problems of shared variance, this item was discarded when computing a total score for the Social Problems subscale of the CBCL and TRF. The internal consistency coefficients of the parent (α = 0.83) and teacher (α = 0.75) versions of the Social Problems subscale were satisfactory in the current study. The Social Competence scale measures the involvement and contribution of the child in sporting clubs and other recreational activities, the number of friends and other social contacts, and the quality of the interpersonal behavior. The *Achenbach System of Empirically Based Assessment* is a reliable, valid, and widely used scale to assess psychopathological symptoms in children and adolescents in the general population as well as in clinical samples [40, 41]. The psychometric properties of the CBCL are also well-established in children and adolescents with ASD [e.g., 42].

In order to measure the level of social anxiety, parents completed a subscale of the *Screen for Child Anxiety and Related Emotional Disorders* (SCARED-71; [43]). Parents rated how often (0 = almost never, 1 = sometimes, and 2 = often) their child experienced social anxiety symptoms (9 items; e.g. “I don’t like to be with people I don’t know well”). According to previous research conducted in non-clinical and clinically referred youths including children and adolescents with ASD, the reliability and validity of the parent version of the SCARED-71 are convincing, and this also applies to the social anxiety subscale [43–45]. In the present study the Cronbach’s alpha was also good (α = 0.89).

### Analyses

In order to evaluate differences across various diagnostic and age groups, 3 (ASD vs. clinical control vs. non-clinical control) × 2 (children vs. adolescents) multivariate analyses of variance (MANOVAs) were conducted on the data of each informant separately (i.e., children/adolescents, parents, and teachers). Given the unequal gender distributions among the three groups, we decided to include this variable as a covariate in the analyses (i.e., MANCOVAs). The primary analysis was the MANCOVA involving youths’ self-report of loneliness on the LACA, which also included the WSIS desire for social contact as dependent variable. The MANCOVA performed on the parent data included SSO social skills, SCARED social anxiety, and the CBCL social problems and social competence scales as dependent variables. The MANCOVA carried out on the teacher data included TRF social problems and SSO social skills as the dependent variables. As the analyses were carried out separately for each informant, the N’s differed from one MANCOVA to another. Furthermore, cases were only included in the MANCOVA when all measures of the pertinent informant were available. Percentages of missing data were 1% for the self-report, 10% for the parent-report, and 27% for the teacher-report scales.

In order to explore the relations between loneliness and the other social variables, partial correlations controlling for gender were calculated for the total sample as well as for the three diagnostic groups separately.

## Results

### Group Comparisons

#### Children and Adolescents’ Self-Report

**Fig. 1:**
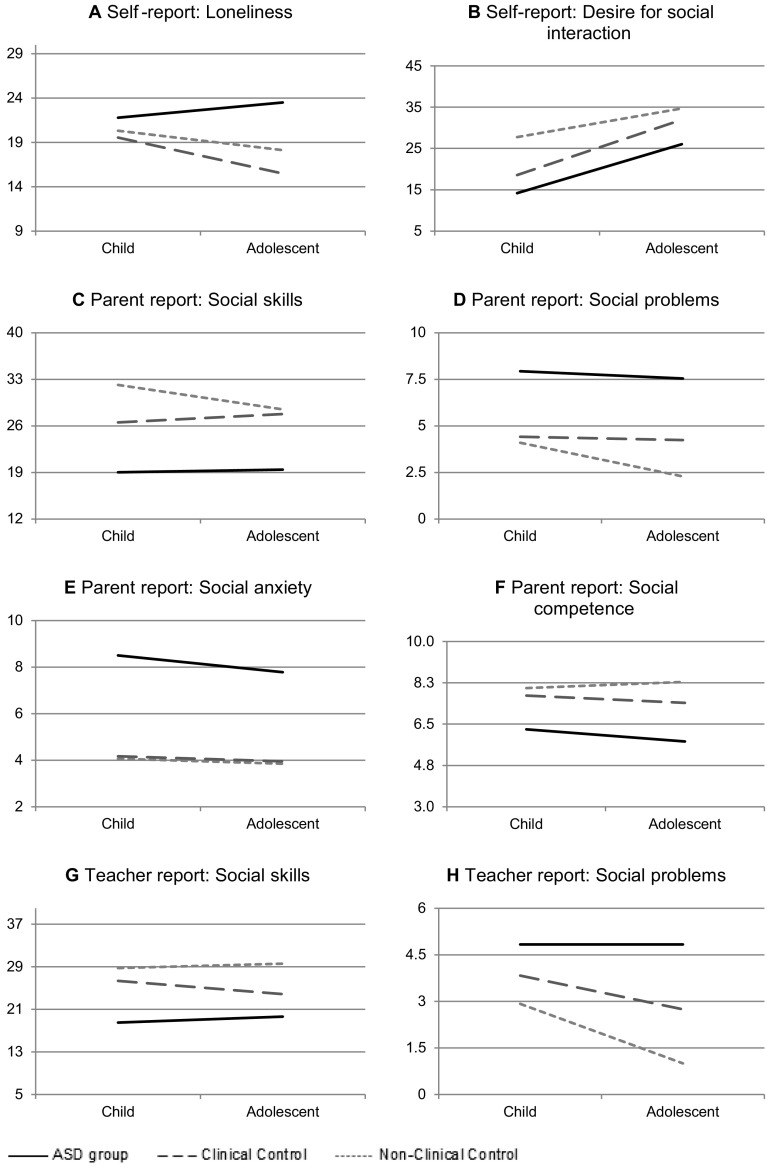
Mean scores on loneliness and other social indices split by diagnostic and age groups

The 3 (diagnostic group: ASD vs. clinical control vs. non-clinical control) × 2 (age group: children vs. adolescents) MANCOVA on self-reported loneliness and desire for social interaction revealed statistically significant main effects of diagnostic group [*F*(4,492) = 14.01, *p* < .001, η^2^ = 0.010], age group [*F*(2,245) = 27.21, *p* < .001, η^2^ = 0.108], and an interaction effect of diagnostic group and age group [*F*(4,492) = 3.10, *p* < .05, η^2^ = 0.030]. Follow-up ANCOVAs showed that the main effects were significant for both the LACA and the WSIS, whereas the interaction effect only attained significance in the case of the LACA (see Table 3) Post-hoc analyses indicated that in general the ASD group displayed higher levels of loneliness as compared to the clinical and non-clinical control groups, while the clinical and non-clinical control group did not statistically differ from each other. Further, in contrast with our hypothesis, children reported significantly higher levels of loneliness than adolescents, although this was only true for the clinical and non-clinical control groups. In the ASD group, no significant difference in loneliness between both age groups was found, although adolescents scored slightly higher than children (see Fig. 1panel a). Note that within the child age group, no statistical differences in loneliness between the three diagnostic groups were noted. However, within the adolescent age group, the three diagnostic groups did significantly differ: the ASD group clearly displayed the highest levels of loneliness, followed by the non-clinical control group and the clinical control group, which exhibited the lowest scores.

**Table 3:** Mean scores (standard deviations) on loneliness and other social variables split by diagnostic and age groups, and results of the follow-up ANCOVAs

Variable	Age group	ASD group	Clinical control	Non-clinical control	Diagnostic group	Age group	Interaction
Self-report					*F*(2,253)	*F*(1,253)	*F*(2,253)
LACA loneliness	Child	21.77 (7.98)_a_	19.54 (7.98)_a_	20.32 (6.14)_a_	12.84***	3.98*	3.77*
	Adolescent	23.50 (7.04)_a_	15.48 (3.35)_b_	18.12 (4.58)_c_			
WSIS desire for interaction	Child	14.15 (10.89)_a_	18.57 (11.49)_a_	27.76 (11.65)_b_	16.82***	51.52***	2.43
	Adolescent	26.04 (12.31)_c_	32.00 (11.87)_d_	34.71 (9.51)_d_			
Parent-report					*F*(2,229)	*F*(1,229)	*F*(2,229)
SSO social skills	Child	19.01 (9.46)_a_	26.50 (8.76)_b_	32.14 (8.12)_c_	24.71***	0.35	1.74
	Adolescent	19.41 (10.31)_a_	27.76 (9.90)_b_	28.46 (8.66)_b_			
CBCL social problems	Child	7.94 (4.28)_a_	4.42 (3.55)_b_	4.10 (3.92)_b_	29.25***	2.89	1.31
	Adolescent	7.55 (3.41)_a_	4.24 (3.63)_b_	2.28 (2.05)_c_			
CBCL social competence	Child	6.28 (2.04)_a_	7.71 (1.74)_b_	8.03 (1.90)_b_	17.33***	0.74	0.65
	Adolescent	5.77 (2.10)_a_	7.40 (2.22)_b_	8.29 (2.17)_b_			
SCARED social anxiety	Child	8.50 (5.20)_a_	4.17 (3.65)_b_	4.06 (3.61)_b_	23.26***	0.73	0.10
	Adolescent	7.77 (5.00)_a_	3.95 (4.16)_b_	3.85 (2.79)_b_			
Teacher-report					*F*(2,185)	*F*(1,185)	*F*(2,185)
SSO social skills	Child	18.51 (7.75)_a_	26.34 (9.23)_b_	28.76 (9.35)_b_	15.27***	0.07	0.56
	Adolescent	19.61 (8.85)_a_	23.87 (10.54)_ab_	29.59 (8.86)_b_			
TRF social problems	Child	4.83 (3.28)_a_	3.83 (2.78)_ab_	2.92 (3.03)_b_	13.60***	4.95^*^	1.65
	Adolescent	4.83 (3.11)_a_	2.74 (2.07)_b_	1.00 (1.91)_c_			

*LACA* loneliness and aloneness in children and adolescents, *WSIS* wish for social interaction scale, *SSO* social skills observation, *CBCL* child behavior checklist, *SCARED* screen for child anxiety related emotional disorders, *TRF* teacher report form. For each variable, within-row and within-column means not sharing similar subscript letters are significantly different at *p* < .05

*p < .05; **p < .01; ***p < .001

As can be seen in Table 3 and Fig. 1 (panel B), the ASD group exhibited lower levels of desire for social interaction than the non-clinical control group, whereas the clinical control group scored in between. Further, in all groups, adolescents had statistically higher levels of desire for social interaction as compared to children.

#### Parent Report

The 3 (diagnostic group) × 2 (age group) MANCOVA only revealed a significant main effect of diagnostic group [*F*(8,440) = 10.95, *p* < .001, η^2^ = 0.017]. As expected, the follow-up ANCOVAs indicated that this was mainly due to the ASD group which deviated on all measures from the two control groups. More precisely, regardless of age, the ASD group scored higher on social problems and social anxiety but lower on social skills and social competence than the clinical and non-clinical control groups (see Table 3and Fig. 1 panels c–f).

#### Teacher Report

The MANCOVA performed on the teacher-report data only revealed a statistically main effect of diagnostic group [*F*(4,356) = 10.15, *p* < .001, η^2^ = 0.010] and a borderline significant effect of age group [*F*(2,177) = 2.86, *p* = .06, η^2^ = 0.003]. Follow-up ANCOVAs revealed that the main effect of diagnostic group showed itself for both teacher-rated social skills and social problems. Post-hoc analyses indicated that teachers provided lower ratings of social skills for children and adolescents in the ASD group than for youths in the clinical and non-clinical control groups (see Table 3panel g). With regard to the teacher-rated social problems, it was found that the ASD group displayed the highest scores, followed by the clinical control group, and the lowest scores for the non-clinical control group. Note further that in general teachers reported lower levels of social problems for adolescents than for children.

### Correlations

Table 4 shows the correlations between loneliness and the other social variables for the total sample and for the three diagnostic groups separately. Within the total sample, loneliness correlated with all other variables. Surprisingly, a negative correlation was found between loneliness and the desire for social interaction, indicating that youth with higher levels of loneliness expressed less desire for social interaction. Further, loneliness was positively associated with parent and teacher ratings of social problems and parent ratings of social anxiety, whereas negative links were noted with parent and teacher ratings of social skills and competence. Other correlations in the total sample were as predicted and suggested that higher levels of social skills and competence were associated with a stronger desire for social interaction, and lower levels of social problems and social anxiety. Note that parent- and teacher-rated social skills and social problems correlated significantly suggesting a convincing interrater agreement.

**Table 4:** Partial correlations (corrected for gender) between loneliness and the other social variables as computed for the total group and the three diagnostic groups separately

	Self-report		Parent-rated				Teacher-rated	
	1	2	3	4	5	6	7	8
1. LACA loneliness								
2. WSIS desire for social interaction								
Total sample	−0.16*							
ASD group	0.06							
Clinical control	−0.13							
Non-clinical control	−0.23*							
3. SSO social Skills								
Total sample	−0.21**	0.17**						
ASD group	−0.04	−0.07						
Clinical control	−0.24*	0.06						
Non-clinical control	−0.13	0.12						
4. CBCL social problems								
Total sample	0.38***	−0.17*	−0.39***					
ASD group	0.13	−0.11	−0.12					
Clinical control	0.35^**^	−0.13	−0.37**					
Non-clinical control	0.47***	0.18	−0.22^*^					
5. CBCL social competence								
Total sample	−0.24***	0.14^*^	0.31^***^	−0.43^***^				
ASD group	−0.25*	0.02	0.14	−0.31*				
Clinical control	−0.03	−0.10	0.22	−0.37**				
Non-clinical control	−0.25*	0.14	0.18	−0.22*				
6. SCARED social anxiety								
Total sample	0.29***	−0.18**	−0.52***	0.42***	−0.30***			
ASD group	0.16	−0.09	−0.47***	0.18	0.04			
Clinical control	0.29*	0.02	−0.46***	0.44***	−0.26*			
Non-clinical control	0.21	−0.12	−0.12	0.24*	−0.27*			
7. SSO social skills								
Total sample	−0.19**	0.21**	0.26**	−0.25**	0.19**	−0.24**		
ASD group	−0.02	0.15	0.14	−0.05	0.01	−0.22		
Clinical control	−0.06	−0.11	−0.02	0.02	0.15	−0.13		
Non-clinical control	−0.21	−0.21	0.07	−0.15	−0.06	0.14		
8. TRF SOCIAL problems								
Total sample	0.29***	−0.18*	−0.12	0.47***	−0.28***	0.10	−0.35***	
ASD group	0.14	0.04	0.21	0.47**	−0.27	−0.10	0.08	
Clinical control	0.21	−0.15	−0.12	0.19	0.03	−0.00	−0.35*	
Non-clinical control	0.37^*^	−0.15	0.05	0.44***	−0.20	−0.04	−0.37**	

*LACA* loneliness and aloneness in children and adolescents, *WSIS* wish for social interaction scale, *SSO* social skills observation, *CBCL* child behavior checklist, *SCARED* screen for child anxiety related emotional disorders, *TRF* teacher report form. Correlations are based on variable numbers of participants: self- and parent-report scales, *n* = 227, self- and teacher-report measures, *n* = 184, and parent- and teacher-report questionnaires, *n* = 169

*p < .05; **p < .01; ***p < .001

The correlations computed for each diagnostic group separately were conducted on smaller numbers of participants and thus obviously subject to power problems. Nevertheless, loneliness showed some anticipated links with the other social variables (see Table 4). In the ASD group, loneliness correlated negatively with parent-rated social competence. Further, in the clinical control group, loneliness was positively related to parent-rated social problems and social anxiety and negatively associated with parent-rated social skills. Finally, in the non-clinical control group, loneliness correlated positively with parent- and teacher-rated social problems and negatively with self-rated desire for social interaction and parent-rated social competence.

## Discussion

In the present study, the phenomenon of loneliness in youths with ASD was examined in more detail. Children and adolescents with ASD completed a self-report questionnaire for measuring this construct and their scores were compared with those of a clinical control group of youths with ADHD and a non-clinical control group of typically developing children and adolescents. In line with the first hypothesis, the ASD group overall reported elevated levels of loneliness as compared to the clinical and non-clinical control groups, but this was mainly true for youths in adolescent age range. In fact, no significant differences between the three diagnostic groups were found in the younger participants. These findings are well in line with previous studies showing that youths with ASD are indeed more prone to feelings of loneliness and that, in comparison with clinical and non-clinical control groups, this problem becomes really manifest during adolescence [14, 16–21].

In contrast to what has been suggested in the loneliness literature (hypothesis 2; [3, 7]), the results of our study demonstrate that adolescents in general displayed lower levels of loneliness than children. One explanation for this unexpected finding is concerned with the fact that loneliness appears to be a multidimensional construct and that various aspects of loneliness may manifest themselves during different developmental stages [3]. In relation to this point, it should be kept in mind that in the present study only one specific dimension of loneliness, namely peer-related loneliness. Maes and colleagues [36] also noted that that this specific aspect of loneliness decreased from childhood to adolescence, whereas other dimensions of loneliness (i.e., parent-related loneliness) increased as youths became older. Another possibility is that children complete a measure like the LACA with a somewhat different perspective than adolescents, resulting in differential loneliness scores for both age groups. Finally, it is also possible that most adolescents are capable of successfully taking the developmental hurdle of connecting to the peer group [46]. It should be borne in mind, however, that most studies on loneliness including the present investigation are cross-sectional in nature, and that more prospective research is necessary to test these possibilities. Interestingly, there is a recent longitudinal trajectory study in non-clinical youths (*N* = 586) by Qualter and colleagues [47] which shows that there are more young people who exhibit stable low (37%) or declining (23%) levels of loneliness for the interval between 7 and 17 years, whereas a minority of 22% and 18% display respectively stable high or increasing levels. It would be interesting to include youths with ASD in such a study and learn more about the developmental pathway of loneliness in youth with this developmental disorder.

In line with our previous study [25], the ASD group exhibited lower levels of self-reported desire for social interaction as compared to the non-clinical control group, while the clinical control group scored in between. Overall, there was a significant effect of age group for this variable, which reflects that adolescents indeed have a stronger wish for social interaction than children [7]. Interestingly, this age effect was not only observable in the clinical and non-clinical control groups, but also in the ASD group, which suggests that even these youngsters have a stronger “wish to belong” during the developmental stage of adolescence.

In keeping with hypothesis 3, statistically significant group differences were found with regard to the parent- and teacher-rated social variables. As expected, the ASD group displayed lower levels of social skills and social competence but higher levels of social problems and social anxiety as compared to the clinical and non-clinical control groups, which is hardly surprising given that deficits in social functioning are one of the defining features of autism spectrum problems [8, 9]. On some of these social variables, the clinical control group also deviated significantly from the non-clinical control group. For example, according to the parents, children and adolescents with ADHD displayed lower levels of social skills and higher levels of social problems than typically developing youths. These findings are in line with previous research demonstrating that ADHD is also to some extent associated with social difficulties [29–31, 48, 49].

The correlational analysis generally showed that the expected links between loneliness and other social variables. That is, negative correlations were found with social skills and social competence, while positive correlations emerged with social problems and social anxiety. These results are well in line with previous research showing that high levels of loneliness are associated with poor social skills, weak social competence [3, 50], and high levels of social anxiety and other social problems [51]. Most of these relationships are thought to be bi-directional in nature, indicating that on the one hand loneliness may be the result of poor social functioning, but that one the other hand loneliness is likely to promote social difficulties [3]. To gain more insight in the role of loneliness in social functioning, especially in youths with ASD, it might be worthwhile to employ the experience sampling method (ESM). ESM repeatedly asks participants to record feelings, cognitions, and behaviors within the context of their daily life, and represents a very informative way to study psychopathology and related phenomena [52].

The correlation between loneliness and the desire for social interaction was negative, which is on first sight surprising as one might expect loneliness to be coupled with a stronger wish to interact with other people. This seems to indicate that the relation between loneliness and desire for social interaction is more complex and probably subject to moderating variables. For example, it may well be that when combined with positive affect, feelings of loneliness may fuel the wish for social interaction, whereas feelings of loneliness which are associated with negative feelings (dysphoria, shyness, or social anxiety) may undermine the desire for social interaction and prompt withdrawal behavior. Other factors possibly involved are the chronicity of loneliness, victimization, children’s self-esteem, and the level of social skills [3, 6].

As noted earlier, one drawback of this study was the cross-sectional design, which not only prevented the investigation of developmental trends but also hindered us in testing causal relationships between loneliness and other social variables. The role of loneliness in the social and emotional well-being of youths with ASD is likely to be rather complex, and requires a more elaborated, longitudinal investigation that should also include other relevant variables such as depression [53]. A further limitation of the present study was the lack of standardized instruments for diagnosing ASD (e.g., Autism Diagnostic Interview-Revised; [54]), ADHD, or comorbid psychopathology (e.g., Structured Clinical Interview for DSM-IV Childhood Disorders; [55]) in the clinical groups. Note in passing that almost one quarter (i.e., 23.3%) of the children and adolescents in the ASD group also had a diagnosis of ADHD, which of course somewhat obscures the comparison with the clinical control group that consisted of youths who had a primary diagnosis of ADHD (but no ASD). The coexistence ASD and ADHD is a common phenomenon [56], and in spite of the fact that we did not control for this diagnostic overlap, we did document significant differences in loneliness and other social variables between these two groups, which of course suggests that the presence of ASD was the determining factor. Another shortcoming pertains to the fact that DSM-IV-TR diagnoses were still used in the present study [8]. Since the majority of the youth in the ASD group were diagnosed with PDD-NOS, it might be that not all participants of the ASD group would fulfill the DSM-5 (*Diagnostic Statistical Manual of Mental Disorders, fifth edition*; [9]) criteria for ASD [57, 58]. Our ASD group can best be considered as a high-functioning ASD group with mild to moderate ASD symptoms in terms of the DSM-5. A final demerit has to do with the unequal gender distribution across the three groups: the ASD group and the clinical control group of youths with ADHD clearly contained more boys than girls [33, 34], whereas in the nonclinical control group the gender distribution was more equal. Available studies suggest that there are no pronounced differences in loneliness between boys and girls [e.g., 36], but it should be admitted that research in clinical samples (such as youths with ASD) is lacking and so this remains a topic of future inquiry.

The results of the present study are also relevant for clinical practice. Loneliness seems to be a rather overlooked problem that deserves more attention. Clinicians should focus more on understanding the implications of social impairments and the unmet desire for social interaction in youths with ASD. These youngsters often have a learning history of social failure and disappointment and might simply give up and choose to protect themselves against future difficulties by means of withdrawal and avoidance behavior, thereby becoming trapped in isolation and feelings of loneliness [59]. Interventions that enhance social skills might be of great value. In fact, it has been demonstrated that social skills training for children with ASD can successfully decrease feelings of loneliness [60]. It is recommended to implement this type of intervention before the transition from primary to secondary school so that children are better equipped to face the social challenges of adolescence.

## Summary

The results of the current study revealed that youths with ASD generally reported higher levels of loneliness as compared to clinical (ADHD) and non-clinical control youths, but this difference was only statistically significant at the adolescent age. Youths with ASD expressed lower levels of desire for social interaction than the control groups, and findings also indicated that even these youngster display an increase in the desire for social interaction during adolescence. Further, as expected, the ASD group clearly displayed lower levels of social competence and social skills and higher levels of social problems and social anxiety as compared to the control groups. These findings seem to fit with the picture that youths with ASD, especially during the adolescent years when they experience at least some desire for social interaction, often fail to realize satisfactory social interactions resulting in feelings of loneliness [17, 25]. This also suggests that social skills training might be particularly valuable in terms of intervention [60]. That is, by increasing the social abilities of young people with ASD, they might be able to adequately meet their limited but still present wish for interacting with others, thereby preventing or alleviating feelings of loneliness.
